# Missing value imputation using least squares techniques in contaminated matrices

**DOI:** 10.1016/j.mex.2022.101683

**Published:** 2022-04-02

**Authors:** Marisol Garcia-Peña, Sergio Arciniegas-Alarcón, Wojtek J. Krzanowski

**Affiliations:** a Pontificia Universidad Javeriana, Departamento de Matemáticas, Bogotá, Colombia; b Universidad de La Sabana, Facultad de Ingeniería, Chía, Colombia; c University of Exeter, College of Engineering, Mathematics and Physical Sciences, Exeter, UK

**Keywords:** Eigenvalues, Eigenvectors, Robust singular value decomposition, Missing values, Iterative computational scheme, Cross-validation, Genotype-by-environment interaction

## Abstract

This paper describes strategies to reduce the possible effect of outliers on the quality of imputations produced by a method that uses a mixture of two least squares techniques: regression and lower rank approximation of a matrix. To avoid the influence of discrepant data and maintain the computational speed of the original scheme, pre-processing options were explored before applying the imputation method. The first proposal is to previously use a robust singular value decomposition, the second is to detect outliers and then treat the potential outliers as missing. To evaluate the proposed methods, a cross-validation study was carried out on ten complete matrices of real data from multi-environment trials. The imputations were compared with the original data using three statistics: a measure of goodness of fit, the squared cosine between matrices and the prediction error. The results show that the original method should be replaced by one of the options presented here because outliers can cause low quality imputations or convergence problems.

• The imputation algorithm based on Gabriel's cross-validation method uses two least squares techniques that can be affected by the presence of outliers. The inclusion of a robust singular value decomposition allows both to robustify the procedure and to detect outliers and consider them later as missing. These forms of pre-processing ensure that the algorithm performs well on any dataset that has a matrix form with suspected contamination.

## Graphical abstract

**Figure:**
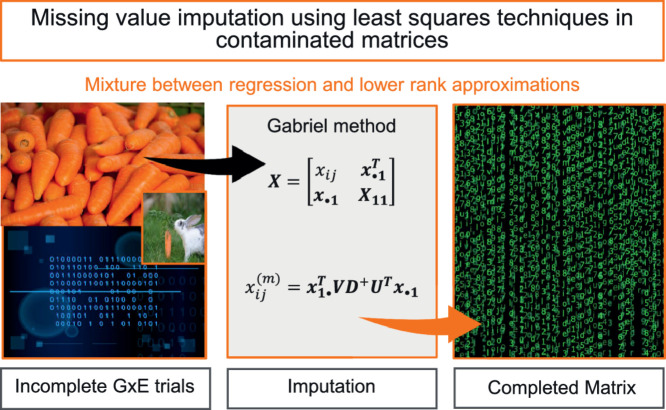

Specifications table

**Table:** 

Subject Area:	Agricultural and Biological Sciences
More specific subject area:	*Biometry; Statistics*
Method name:	*Gabriel cross-validation method*
Name and reference of original method:	*K.R. Gabriel. Le biplot – outil d'exploration de données multidimensionnelles. Journal de la Societé Française de Statistique. (143) (2002) 5-55.* *S. Arciniegas-Alarcón, M. García-Peña, W.J. Krzanowski, C.T.S Dias. An alternative methodology for imputing missing data in trials with genotype-by-environment interaction: some new aspects. Biometrical Letters. (51) (2014) 75-88.*
Resource availability:	*There are no special resources*

## Method details

In 2002, Ruben Gabriel proposed a regression algorithm for cross-validation of the low-rank approximations of any complete data matrix and used it to present several important aspects of the biplot, a powerful and well-known tool for multivariate statistical analysis using the rank two approximations of any matrix [1]. The algorithm is based on the singular value decomposition (SVD) of a matrix, and was applied in different areas such as plant breeding by Dias and Krzanowski [2] to find the most parsimonious additive main effects with multiplicative interaction model (AMMI) and thus explain as best as possible the genotype x environment interaction (G×E) [3].

The algorithm belongs to a class of methods known as leave-one-out, which consist of eliminating each element of the study matrix and producing a prediction using the remaining data [4]. This feature was used by Perry [5] and Owen and Perry [6] to generalise the algorithm and more efficiently perform what they call the bi-cross-validation of an SVD. In that research it was proposed to eliminate sub-matrices instead of a simple element, obtaining a leave-group-out method; the computational implementation is available in the *bcv* package of the statistical environment R [7]. Recently, Fu and Perry [8] used Gabriel's method to determine the number of clusters in unsupervised learning for high dimensional data.

The predictive properties of Gabriel's method can also serve as a basis for handling missing information. Arciniegas-Alarcón et al. [9] proposed an iterative extension of the algorithm to obtain a deterministic imputation system in multi-environment trials and called it the GabrielEigen system because the method uses SVD eigenvectors and eigenvalues to make the imputations of missing data. Later, Arciniegas-Alarcón et al. [19] generalised the iterative extension by including weights in the imputation equation (WGabriel) and found the weights that optimized the predictive quality of data in an incomplete matrix. Including weights allows for simple and multiple (MI) imputation. Both Gabriel's original method and WGabriel were recently evaluated by Hadasch et al. [11] in the choice of AMMI models and GGE models (Genotype and Genotype x Environment interaction). Finally, alternatives for MI and confidence intervals for missing values computationally more efficient than WGabriel were proposed by García-Peña et al. [12] from GabrielEigen.

To the best of our knowledge, missing data imputation using the Gabriel (or GabrielEigen) method has not been studied in the presence of outliers, and given that the method relies on a least squares technique such as SVD, outliers can decrease the quality of imputations [13]. For this reason, we propose to modify GabrielEigen taking into account two possibilities from the statistical literature: i) Robust GabrielEigen using a robust SVD (rSVD) or ii) Make imputation with GabrielEigen on a pre-processed study matrix. Pre-processing consists of initially detecting outliers by different methods and later considering them as missing [14].

## GabrielEigen method

Suppose that the (*n × p*) matrix **X** contains elements *x_ij_* (i=1,...,n; j=1,...,p), some of which are missing. The rows represent genotypes and the columns the environments. Step 1: Start by inserting into each missing entry the mean of its column, thereby obtaining a completed matrix **X**. Step 2: The columns of **X** are standardised by subtracting *m_j_* from each element and dividing the result by *s_j_* (where *m_j_* and *s_j_* are respectively the mean and the standard deviation of the *j*th column). Step 3: Using the standardised matrix, each original missing entry *x_ij_* is replaced by

(1) $$x_{ij}^{\left(m\right)}=x_{1•}^{T}VD^{+}U^{T}x_{•1}$$

where $\mathrm{UD}V^{T}$ represents the SVD of $X_{11}$ and $D^{+}$ is the Moore-Penrose generalised inverse of ***D.***

Here the vectors $x_{1•}^{T}$, $x_{•1}$ and the matrices V, D and U are obtained from the partition

(2) $$X=\left[\begin{array}{cc} x_{ij} & x_{•1}^{T}\\ x_{•1} & X_{11} \end{array}\right]$$

with $X_{11}=\sum_{k=1}^{m}u_{\left(k\right)}d_{k}v_{\left(k\right)}^{T}=\mathrm{UD}V^{T}$, where $U=[u_{1},u_{2},...,u_{m}]$, $V=[v_{1},v_{2},...,v_{m}]$, $D=diag(d_{1},...,d_{m})$ and m≤min{n−1,p−1}. Note also that for each missing observation the components of the considered partition will be different, and this partition is obtained through elementary operations on the rows and columns of **X**. Step 4**:** This imputation process depends on the choice of the value for *m* in Step 3 and it is usual to choose *m* to be the smallest value satisfying

(3) $$\frac{\sum_{k=1}^{m}d_{k}^{2}}{\sum_{k=1}^{\min\left\{n-1,p-1\right\}}d_{k}^{2}}\geq 0.75.$$

Step 5: Finally, the imputed values must be returned to their original scale, $x_{ij}=m_{j}+s_{j}{\hat{x}}_{ij}^{\left(m\right)}$, replacing them in the matrix **X**. Steps 2 to 5 are then iterated until the imputations achieve stability. This process assumes that *n*>*p.* If this is not the case, then the matrix should first be transposed before conducting the iterations.

## Proposed modifications

To avoid the influence of outliers, two possibilities were considered: Robustify the algorithm or pre-process the data matrix before making the imputation. The first attempt to robustify GabrielEigen consisted of using an rSVD on $X_{11}$ of Eq. (1) and then using the quartile method to detect the outliers and replace them with trimmed means on the vectors $x_{1•}^{T}$ and $x_{•1}$. Also, classic standardisation was replaced by a robust standardisation in the iterative scheme. Although this type of procedure provides robust imputations, the algorithm loses one of its main features, that is, it becomes computationally very intensive, and it would only be worth using for matrices of size (20×5) or smaller. To robustify the imputation and maintain computational speed we tried a two-stage process. This process (TwoStagesG) consists of applying the rSVD reported by García-Peña et al. [13] on the incomplete matrix X, obtaining a robust lower rank approximation $X_{rob}$ and then refining the imputations by applying GabrielEigen on $X_{rob}$. The additional information section (below) describes each step needed to obtain an rSVD of any data matrix.

Another option to avoid possible imputation problems due to discrepant data is to pre-process the X matrix by detecting outliers in the observed information, treating them as missing, and performing the imputation with GabrielEigen on the resulting matrix $X_{pre}$. In this case, the imputation by GabrielEigen will necessarily depend on the outlier detection method and here the following options were considered:

1) QuartileG: This method finds univariate outliers using the quartile method in each of the columns of X. Once the outliers are detected, they are treated as missing and finally GabrielEigen is applied to obtain the required imputations. The quartile method is implemented in the *StatMeasures* package of the statistical environment R [7] and treats as an outlier any value that is greater than $P_{75}+1.5\left(IQR\right)$ or less than $P_{25}-1.5\left(IQR\right)$, where $P_{i}$ and IQR represent the i-th percentile and the interquartile distance, respectively.
2) ColGabriel: This method detects the outliers using a modification of the procedure suggested by Maronna and Yohai [15], treats them as missing and applies the GabrielEigen algorithm to obtain the required imputations. The detection of outliers is done as follows on the incomplete matrix X: i) The rSVD of X is calculated and a robust lower rank approximation, $X_{RLR}$, with *m* components is obtained. To determine *m*, Eq. (3) can be used on the eigenvalues obtained with the rSVD. ii) Missing values in X are filled with any arbitrary value (eg, the median of the values observed in each column), obtaining a $X_{ini}\;$matrix. iii) The $X_{ini}$ SVD is calculated and the imputations are updated with the corresponding positions of a lower rank approximation using *m* components of the SVD, obtaining a new $X_{upd}$ matrix. iv) Setting $X_{ini}=X_{upd}$, step iii) is applied iteratively until some pre-specified level of stability is reached. v) After achieving stability, the lower rank approximation of $X_{upd}$ is found using the SVD with *m* components, ie $X_{LR}$. vi) The least squares $X-X_{LR}$ residuals are calculated and then the absolute value of each residual ($r_{ij}$) is obtained. The matrix of absolute values of the residuals will be denoted by R. vii) The quantile 0.75 of each column of R is calculated and is denoted by $S_{j}$. viii) The robust residuals $r_{ij}\left(rob\right)$ are obtained from the matrix operation $X-X_{RLR}$. ix) If any $|r_{ij}\left(rob\right)|$ is greater than 2.5$S_{j}$, the position (i,j) of X is considered to be a potential outlier.
3) RowGabriel: This method follows the same reasoning as ColGabriel, but in step vii) the quantile 0.75 of each row of R is calculated and is denoted by $S_{i}$. This change means that in step ix) position (i,j) is potentially an outlier if any $|r_{ij}\left(rob\right)|$ is greater than 2.5$S_{i}$.

## Validation of the methods

To test the modifications proposed above, we considered a total of seven imputation methods: GabrielEigen, TwoStagesG, QuartileG, ColGabriel, RowGabriel, EM-AMMI0 and EM-AMMI1. The EM-AMMI method is considered in the literature as a classic method to solve the lack of balance problem in experimental matrices from multi-environment trials. The method mixes the Expectation-Maximization (EM) algorithm with the AMMI model so is called EM-AMMIk where *k* (greater than or equal to zero) is the number of multiplicative components needed to explain the G×E interaction. For further details see Gauch and Zobel [16]. Following the recommendations of Piepho [17] and more recently of Paderewski and Rodrigues [18] and Arciniegas-Alarcón et al. [10] the best results are usually obtained with *k* = 0,1.

To evaluate the methods, ten complete open access datasets from G×E trials were chosen. Table 1 presents the basic information of each one along with the corresponding reference for additional information. In each experiment, the most adequate AMMI model was found by the Eigenvector method [20] to establish what type of interaction it presents. We consider a simple interaction to be that which can be explained with an AMMI1 model, intermediate, that which can be explained with an AMMI2 model, and complex, that which can be explained with models with more than two multiplicative components. Here it is worth mentioning that the literature has already shown that imputation errors using AMMI models increase as the number of components increases, so in this type of experiments it may be that an incomplete matrix can provide the best imputations with an AMMI0 model, but this model it will not necessarily be the same model for further analysis.

**Table 1:** Datasets chosen to perform the cross-validation study.

References	Species	No. of genotypes	No. of environments	Response variable	AMMI model to explain the interaction G×E
Yan et al. [22]	Wheat	18	9	Mean yield	AMMI2
Lavoranti [23]	Eucalyptus	20	7	Mean tree height	AMMI2
Calinski et al. [24]	Pea	18	9	Mean yield	AMMI1
Calinski et al. [25]	Rye	18	15	Mean yield	AMMI2
Farias [26]	Cotton	15	27	Mean yield	AMMI1
Filho et al. [27]	Cotton	17	23	Mean yield	AMMI5
Flores et al. [28]	Bean	15	12	Mean yield	AMMI4
Mattos et al. [29]	Sugarcane	22	5	Mean yield	AMMI1
Rad et al. [30]	Wheat	36	6	Mean yield	AMMI3
Yang [31]	Barley	6	18	Yields	AMMI1

A cross-validation study was carried out on each dataset, initially producing incomplete and contaminated matrices as follows. In each experimental matrix (Y), data were removed according to a missing not at random mechanism – MNAR in two percentages: 10 and 20% with three percentages of outliers: 0, 2 and 4%. The MNAR mechanism is very common in multienvironment trials and the strategy described by Arciniegas-Alarcón et al. [21], which consists of finding the 10th (or 20th) percentile in each environment or column of Y and subsequently treating as missing any data value less than or equal to that percentile. On the remaining information in the incomplete matrix, some positions were randomly contaminated depending on the respective percentage using the distribution $N(\mu_{jEnv}+100\sigma_{jEnv}^{2},\sigma_{jEnv}^{2})$, where $\mu_{jEnv}$ and $\sigma_{jEnv}^{2}$ represent the mean and variance of j-th column (or j-th environment) of the values that were not removed [13].

For each incomplete and contaminated $Y_{IC}$ matrix, each non-missing value was removed in turn and imputed with each of the seven methods and the corresponding imputation was registered in a matrix I of the same dimension as Y. In the positions that were already missing the imputation provided by each system on the $Y_{IC}$ matrix was recorded. Performing this cross-validation, a matrix I was obtained in each combination of percentages of missing data and outliers, and ***I*** was compared with the original matrix Y using three statistics: the prediction error, $P_{e}$; the squared cosine between the two matrices, $GF_{2}=cos^{2}\left(Y,I\right)$; and a measure of fit of the magnitudes between the imputations and the original values, $GF_{1}=1-∥Y-I{∥}^{2}/∥Y{∥}^{2}$. A good imputation method should have the smallest $P_{e}$, and $GF_{1}$ and $GF_{2}$ close to 1. In the case of $GF_{1}$, the statistic can take negative values, indicating that the imputations obtained in I are of such low quality as if it were an array of zeros.

Table 2 presents a summary of the cross-validation study and the specific results for each of the considered matrices can be found in the supplementary material. Table 2 shows that in matrices with 10% missing and without contamination, the best method was the EM-AMMI in six data sets and when the removal percentage was increased to 20%, the number of sets with excellent performance for this method has halved (ie, three). In scenarios without contamination, both GabrielEigen and the proposals described in this paper were very competitive with the classic method. This situation may indicate the existence of outliers in the original and complete data.

**Table 2:** Summary of cross-validation study.

Dataset	Missing	Outliers	Method(s) with lowest $P_{e}$ and biggest $GF_{1}$ and $GF_{2}$	Dataset	Missing	Outliers	Method(s) with lowest $P_{e}$ and biggest $GF_{1}$ and $GF_{2}$
Yan et al. [22]	10%	0%	EM-AMMI0	Filho et al. [27]	10%	0%	EM-AMMI0
	10%	2%	RowGabriel		10%	2%	RowGabriel
	10%	4%	RowGabriel		10%	4%	QuartileG-Col(Row)Gabriel
	20%	0%	EM-AMMI0		20%	0%	EM-AMMI0
	20%	2%	TwoStagesG		20%	2%	ColGabriel
	20%	4%	TwoStagesG		20%	4%	QuartileG-Col(Row)Gabriel
Lavoranti [23]	10%	0%	EM-AMMI0	Flores et al. [28]	10%	0%	EM-AMMI0
	10%	2%	TwoStagesG		10%	2%	RowGabriel
	10%	4%	TwoStagesG		10%	4%	Col(Row)Gabriel
	20%	0%	EM-AMMI0		20%	0%	TwoStagesG
	20%	2%	TwoStagesG		20%	2%	TwoStagesG
	20%	4%	TwoStagesG		20%	4%	TwoStagesG
Calinski et al. [24]	10%	0%	EM-AMMI0	Mattos et al. [29]	10%	0%	TwoStagesG
	10%	2%	TwoStagesG		10%	2%	TwoStagesG
	10%	4%	TwoStagesG		10%	4%	QuartileG
	20%	0%	TwoStagesG		20%	0%	RowGabriel
	20%	2%	TwoStagesG		20%	2%	Col(Row)Gabriel
	20%	4%	TwoStagesG		20%	4%	QuartileG
Calinski et al. [25]	10%	0%	EM-AMMI1	Rad et al. [30]	10%	0%	GabrielEigen
	10%	2%	Col(Row)Gabriel		10%	2%	ColGabriel
	10%	4%	Col(Row)Gabriel		10%	4%	ColGabriel
	20%	0%	EM-AMMI1		20%	0%	QuartileG
	20%	2%	Col(Row)Gabriel		20%	2%	QuartileG
	20%	4%	Col(Row)Gabriel		20%	4%	QuartileG
Farias [26]	10%	0%	GabrielEigen-QuartileG	Yang [31]	10%	0%	EM-AMMI0
	10%	2%	QuartileG-RowGabriel		10%	2%	RowGabriel
	10%	4%	QuartileG-Col(Row)Gabriel		10%	4%	ColGabriel
	20%	0%	QuartileG		20%	0%	QuartileG
	20%	2%	QuartileG		20%	2%	QuartileG
	20%	4%	QuartileG-Col(Row)Gabriel		20%	4%	ColGabriel

Although the situation described above is highlighted, the most important result is found in all situations involving some level of contamination (2 or 4%). In these cases, at least one of the four proposals TwoStagesG, Col(Row)Gabriel and QuartileG always surpasses both the EM-AMMI method and the original GabrielEigen system, regardless of the type of interaction (simple, intermediate or complex) that is shown in Table 1. Furthermore, in some datasets, several proposals presented the same results (see supplementary material). For example, in the Farias [26] dataset with 20% of missing and 4% outliers we see QuartileG - Col(Row)Gabriel, which indicates that QuartileG, ColGabriel and RowGabriel detected the same outliers and for this reason the imputation provided the same results.

## Comments

The four proposals TwoStagesG, ColGabriel, RowGabriel and QuartileG performed well when compared to the classic EM-AMMI and the simple GabrielEigen methods. In the event of contamination in incomplete G×E trials, it is recommended to use one of our proposals and not to use either EM-AMMI or GabrielEigen. In the supplementary material it can be seen that EM-AMMI and GabrielEigen had the highest $P_{e}$ in addition to negative $GF_{1}$ and low $GF_{2}$, which indicates that with outliers the quality of imputations is very poor.

In this study the proposals worked very well, but further research will be needed to determine which procedure might be more efficient: i) Without applying outlier detection as with TwoStagesG or ii) Detecting outliers with any of the other three methods. Until this research is carried out, we can consider the four methods as equivalent and any one can be applied in incomplete G×E trials if there is any suspicion of contamination.

## Appendix Supplementary materials

Image, application 1
