# Supplementary material for: Missing value imputation using least squares techniques in contaminated matrices
Source: MethodsX. 2022 Apr 2;9:101683. doi: 10.1016/j.mex.2022.101683 (PMC9036115; doi:10.1016/j.mex.2022.101683)
Supplement: Supplementary file 1 [file mmc1.docx]

**Additional information**

**Robust singular value decomposition – rSVD (García-Peña et al. 2021)**

Consider a matrix $\boldsymbol{X}$ $\left( n\times p \right)$ with possible missing entries. i) Using the observed information, calculate the vectors of trimmed means (at 10% or 20%) by columns and by rows, giving $\boldsymbol{b}_{\mathbf{1}}$ $\left( 1\times p \right)$ and $\boldsymbol{a}_{\mathbf{1}}$ $\left( n\times1 \right)$ respectively. ii) Determine the presence of outliers in vector $\boldsymbol{a}_{\mathbf{1}}$ by any technique that is preferred for univariate outliers (for example, the quartile method) and if there is any discrepant value replace it with a trimmed mean of the elements of $\boldsymbol{a}_{\mathbf{1}}$. iii) Update the elements of $\boldsymbol{b}_{\mathbf{1}}$ and $\boldsymbol{a}_{\mathbf{1}}$ as follows: $b_{c}=med\left\{ \left| {x_{r,c}}/{a_{r}} \right|;r=1,\ldots,n \right\}$ and $a_{r}=med\left\{ \left| {x_{r,c}}/{b_{c}} \right|;c=1,\ldots,p \right\}$. Go back to step ii) with the vectors $\boldsymbol{b}_{\mathbf{1}}$ and $\boldsymbol{a}_{\mathbf{1}}$ updated and make a new update of the elements of the vectors according to step iii) until you reach some specified convergence criterion. Once the stability of the $\boldsymbol{b}_{\mathbf{1}}$ and $\boldsymbol{a}_{\mathbf{1}}$ vectors is achieved, a robust lower rank approximation of $\boldsymbol{X}$ is obtained by means of the $\boldsymbol{a}_{\mathbf{1}}\boldsymbol{b}_{\mathbf{1}}^{\boldsymbol{T}}$ product whose signs of the elements match those of $\boldsymbol{X}$. To obtain the first singular value and the first right and left singular vector of the robust SVD apply standard SVD to $\boldsymbol{a}_{\mathbf{1}}\boldsymbol{b}_{\mathbf{1}}^{\boldsymbol{T}}$ and record both the first singular value and the first singular right and left vector. To obtain the second singular value and the second right and left vector, follow the same procedure as above, but instead of $\boldsymbol{X}$ as initial matrix use the deflated matrix $\boldsymbol{X}-\boldsymbol{a}_{\mathbf{1}}\boldsymbol{b}_{\mathbf{1}}^{\boldsymbol{T}}$. This iterative strategy, deflating the matrix continues step by step until the desired number of components have been reached.

**Supplementary material**

The values in bold in each table represent the methods with maximum ${GF}_{2}={Cos}^{2}$, ${GF}_{1}$ and minimum prediction error ($P_{e}$)

Table S1: Cross-validation study in Yan et al. (2007)

|  | Dataset: Yan et al. (2007) | | | | | | |
| --- | --- | --- | --- | --- | --- | --- | --- |
| Methods | Missing 10% and Outliers = 0% | | | Methods | Missing 20% and Outliers = 0% | | |
|  | ${Cos}^{2}$ | ${GF}_{1}$ | $P_{e}$ |  | ${Cos}^{2}$ | ${GF}_{1}$ | $P_{e}$ |
| GabrielEigen | 0.9864 | 0.9855 | 0.5179 | GabrielEigen | 0.9841 | 0.9815 | 0.5849 |
| TwoStagesG | 0.9859 | 0.9851 | 0.5254 | TwoStagesG | 0.9867 | 0.9851 | 0.5246 |
| QuartileG | 0.9850 | 0.9836 | 0.5504 | QuartileG | 0.9838 | 0.9814 | 0.5864 |
| ColGabriel | 0.9859 | 0.9851 | 0.5252 | ColGabriel | 0.9852 | 0.9827 | 0.5650 |
| RowGabriel | 0.9860 | 0.9849 | 0.5276 | RowGabriel | 0.9840 | 0.9820 | 0.5777 |
| **EM-AMMI0** | **0.9898** | **0.9896** | **0.4386** | **EM-AMMI0** | **0.9881** | **0.9866** | **0.4984** |
| EM-AMMI1 | 0.9868 | 0.9863 | 0.5040 | EM-AMMI1 | 0.9836 | 0.9818 | 0.5803 |
|  | Missing 10% and Outliers = 2% | | |  | Missing 20% and Outliers = 2% | | |
| GabrielEigen | 0.9010 | 0.8583 | 1.6191 | GabrielEigen | 0.9126 | 0.8754 | 1.5183 |
| TwoStagesG | 0.9850 | 0.9836 | 0.5502 | **TwoStagesG** | **0.9858** | **0.9838** | **0.5473** |
| QuartileG | 0.9852 | 0.9838 | 0.5470 | QuartileG | 0.9838 | 0.9813 | 0.5875 |
| ColGabriel | 0.9862 | 0.9852 | 0.5235 | ColGabriel | 0.9845 | 0.9821 | 0.5756 |
| **RowGabriel** | **0.9863** | **0.9854** | **0.5205** | RowGabriel | 0.9823 | 0.9783 | 0.6338 |
| EM-AMMI0 | 0.9289 | 0.9042 | 1.3310 | EM-AMMI0 | 0.9473 | 0.9269 | 1.1627 |
| EM-AMMI1 | 0.0307 | -16.3887 | 17.9339 | EM-AMMI1 | 0.0880 | -7.5707 | 12.5907 |
|  | Missing 10% and Outliers = 4% | | |  | Missing 20% and Outliers = 4% | | |
| GabrielEigen | 0.9544 | 0.9082 | 1.3031 | GabrielEigen | 0.9290 | 0.8771 | 1.5079 |
| TwoStagesG | 0.9848 | 0.9835 | 0.5531 | **TwoStagesG** | **0.9859** | **0.9838** | **0.5473** |
| QuartileG | 0.9854 | 0.9840 | 0.5435 | QuartileG | 0.9832 | 0.9810 | 0.5935 |
| ColGabriel | 0.9862 | 0.9853 | 0.5211 | ColGabriel | 0.9818 | 0.9777 | 0.6428 |
| **RowGabriel** | **0.9863** | **0.9855** | **0.5173** | RowGabriel | 0.9817 | 0.9777 | 0.6425 |
| EM-AMMI0 | 0.9207 | 0.8614 | 1.6010 | EM-AMMI0 | 0.9452 | 0.9138 | 1.2630 |
| EM-AMMI1 | 0.0713 | -45.4399 | 29.3081 | EM-AMMI1 | 0.0407 | -13.6743 | 16.4748 |

Table S2: Cross-validation study in Lavoranti (2003)

|  | Dataset: Lavoranti (2003) | | | | | | |
| --- | --- | --- | --- | --- | --- | --- | --- |
| Methods | Missing 10% and Outliers = 0% | | | Methods | Missing 20% and Outliers = 0% | | |
|  | ${Cos}^{2}$ | ${GF}_{1}$ | $P_{e}$ |  | ${Cos}^{2}$ | ${GF}_{1}$ | $P_{e}$ |
| GabrielEigen | 0.9964 | 0.9963 | 1.0593 | GabrielEigen | 0.9961 | 0.9958 | 1.1274 |
| TwoStagesG | 0.9971 | 0.9970 | 0.9555 | TwoStagesG | 0.9965 | 0.9963 | 1.0548 |
| QuartileG | 0.9961 | 0.9959 | 1.1047 | QuartileG | 0.9960 | 0.9956 | 1.1442 |
| ColGabriel | 0.9966 | 0.9965 | 1.0314 | ColGabriel | 0.9960 | 0.9957 | 1.1398 |
| RowGabriel | 0.9967 | 0.9965 | 1.0240 | RowGabriel | 0.9962 | 0.9958 | 1.1223 |
| **EM-AMMI0** | **0.9971** | **0.9971** | **0.9397** | **EM-AMMI0** | **0.9970** | **0.9968** | **0.9880** |
| EM-AMMI1 | 0.9934 | 0.9933 | 1.4160 | EM-AMMI1 | 0.9946 | 0.9943 | 1.3039 |
|  | Missing 10% and Outliers = 2% | | |  | Missing 20% and Outliers = 2% | | |
| GabrielEigen | 0.9686 | 0.9432 | 4.1315 | GabrielEigen | 0.9595 | 0.9320 | 4.5191 |
| **TwoStagesG** | **0.9970** | **0.9969** | **0.9616** | **TwoStagesG** | **0.9964** | **0.9962** | **1.0702** |
| QuartileG | 0.9962 | 0.9960 | 1.0923 | QuartileG | 0.9960 | 0.9956 | 1.1503 |
| ColGabriel | 0.9965 | 0.9964 | 1.0431 | ColGabriel | 0.9962 | 0.9958 | 1.1266 |
| RowGabriel | 0.9550 | 0.9459 | 4.0306 | RowGabriel | 0.9962 | 0.9958 | 1.1266 |
| EM-AMMI0 | 0.8625 | 0.7775 | 8.1769 | EM-AMMI0 | 0.8012 | 0.6463 | 10.3100 |
| EM-AMMI1 | 0.0388 | -32.3967 | 100.1788 | EM-AMMI1 | 0.1579 | -7.9278 | 51.7960 |
|  | Missing 10% and Outliers = 4% | | |  | Missing 20% and Outliers = 4% | | |
| GabrielEigen | 0.9823 | 0.9510 | 3.8377 | GabrielEigen | 0.9817 | 0.9470 | 3.9905 |
| **TwoStagesG** | **0.9970** | **0.9969** | **0.9719** | **TwoStagesG** | **0.9967** | **0.9964** | **1.0438** |
| QuartileG | 0.9962 | 0.9961 | 1.0814 | QuartileG | 0.9959 | 0.9955 | 1.1578 |
| ColGabriel | 0.9965 | 0.9964 | 1.0438 | ColGabriel | 0.9961 | 0.9957 | 1.1308 |
| RowGabriel | 0.9605 | 0.9531 | 3.7528 | RowGabriel | 0.9961 | 0.9957 | 1.1308 |
| EM-AMMI0 | 0.8652 | 0.7595 | 8.5015 | EM-AMMI0 | 0.8095 | 0.6406 | 10.3930 |
| EM-AMMI1 | 0.0443 | -53.8285 | 128.3594 | EM-AMMI1 | 0.1345 | -18.5977 | 76.7408 |

Table S3: Cross-validation study in Calinski et al. (2009a)

|  | Dataset: Calinski et al. (2009a) | | | | | | |
| --- | --- | --- | --- | --- | --- | --- | --- |
| Methods | Missing 10% and Outliers = 0% | | | Methods | Missing 20% and Outliers = 0% | | |
|  | ${Cos}^{2}$ | ${GF}_{1}$ | $P_{e}$ |  | ${Cos}^{2}$ | ${GF}_{1}$ | $P_{e}$ |
| GabrielEigen | 0.9954 | 0.9953 | 3.2788 | GabrielEigen | 0.9954 | 0.9948 | 3.4193 |
| TwoStagesG | 0.9954 | 0.9953 | 3.2622 | **TwoStagesG** | **0.9956** | **0.9951** | **3.3276** |
| QuartileG | 0.9954 | 0.9952 | 3.2877 | QuartileG | 0.9954 | 0.9948 | 3.4393 |
| ColGabriel | 0.9954 | 0.9953 | 3.2718 | ColGabriel | 0.9955 | 0.9950 | 3.3532 |
| RowGabriel | 0.9955 | 0.9954 | 3.2405 | RowGabriel | 0.9956 | 0.9951 | 3.3397 |
| **EM-AMMI0** | **0.9957** | **0.9956** | **3.1605** | EM-AMMI0 | 0.9955 | 0.9950 | 3.3525 |
| EM-AMMI1 | 0.9903 | 0.9900 | 4.7507 | EM-AMMI1 | 0.9914 | 0.9905 | 4.6440 |
|  | Missing 10% and Outliers = 2% | | |  | Missing 20% and Outliers = 2% | | |
| GabrielEigen | 0.4749 | -1.3976 | 73.7379 | GabrielEigen | 0.6200 | -0.2155 | 52.5027 |
| **TwoStagesG** | **0.9956** | **0.9954** | **3.2434** | **TwoStagesG** | **0.9954** | **0.9949** | **3.3866** |
| QuartileG | 0.9955 | 0.9953 | 3.2557 | QuartileG | 0.9953 | 0.9947 | 3.4783 |
| ColGabriel | 0.9955 | 0.9954 | 3.2470 | ColGabriel | 0.9954 | 0.9948 | 3.4317 |
| RowGabriel | 0.9955 | 0.9954 | 3.2470 | RowGabriel | 0.9954 | 0.9948 | 3.4317 |
| EM-AMMI0 | 0.6410 | -0.3236 | 54.7880 | EM-AMMI0 | 0.6910 | 0.0215 | 47.1070 |
| EM-AMMI1 | 0.0016 | -2572.29 | 2415.74 | EM-AMMI1 | 0.0091 | -801.15 | 1348.76 |
|  | Missing 10% and Outliers = 4% | | |  | Missing 20% and Outliers = 4% | | |
| GabrielEigen | 0.8111 | -0.1353 | 50.7408 | GabrielEigen | 0.7853 | -0.3006 | 54.3098 |
| **TwoStagesG** | **0.9958** | **0.9955** | **3.1792** | **TwoStagesG** | **0.9953** | **0.9947** | **3.4572** |
| QuartileG | 0.9954 | 0.9953 | 3.2787 | QuartileG | 0.9950 | 0.9943 | 3.5829 |
| ColGabriel | 0.9955 | 0.9953 | 3.2692 | ColGabriel | 0.9951 | 0.9944 | 3.5545 |
| RowGabriel | 0.9955 | 0.9953 | 3.2692 | RowGabriel | 0.9951 | 0.9944 | 3.5545 |
| EM-AMMI0 | 0.6195 | -1.0258 | 67.7801 | EM-AMMI0 | 0.6424 | -0.8533 | 64.8299 |
| EM-AMMI1 | 0.0336 | -5115.15 | 3406.26 | EM-AMMI1 | 0.0048 | -9106.87 | 4544.80 |

Table S4: Cross-validation study in Calinski et al. (2009b)

|  | Dataset: Calinski et al. (2009b) | | | | | | |
| --- | --- | --- | --- | --- | --- | --- | --- |
| Methods | Missing 10% and Outliers = 0% | | | Methods | Missing 20% and Outliers = 0% | | |
|  | ${Cos}^{2}$ | ${GF}_{1}$ | $P_{e}$ |  | ${Cos}^{2}$ | ${GF}_{1}$ | $P_{e}$ |
| GabrielEigen | 0.9972 | 0.9972 | 4.1935 | GabrielEigen | 0.9966 | 0.9965 | 4.6750 |
| TwoStagesG | 0.9969 | 0.9969 | 4.4000 | TwoStagesG | 0.9966 | 0.9964 | 4.7170 |
| QuartileG | 0.9971 | 0.9971 | 4.2404 | QuartileG | 0.9966 | 0.9964 | 4.7172 |
| ColGabriel | 0.9972 | 0.9972 | 4.1613 | ColGabriel | 0.9966 | 0.9964 | 4.6996 |
| RowGabriel | 0.9971 | 0.9971 | 4.2128 | RowGabriel | 0.9966 | 0.9965 | 4.6603 |
| EM-AMMI0 | 0.9968 | 0.9968 | 4.4640 | EM-AMMI0 | 0.9964 | 0.9961 | 4.8798 |
| **EM-AMMI1** | **0.9974** | **0.9973** | **4.0647** | **EM-AMMI1** | **0.9969** | **0.9966** | **4.5546** |
|  | Missing 10% and Outliers = 2% | | |  | Missing 20% and Outliers = 2% | | |
| GabrielEigen | 0.3821 | -4.8475 | 189.9559 | GabrielEigen | 0.2397 | -12.8360 | 292.1957 |
| TwoStagesG | 0.9968 | 0.9967 | 4.4848 | TwoStagesG | 0.9965 | 0.9963 | 4.7589 |
| QuartileG | 0.9972 | 0.9972 | 4.1899 | QuartileG | 0.9965 | 0.9963 | 4.7508 |
| **ColGabriel** | **0.9972** | **0.9972** | **4.1898** | **ColGabriel** | **0.9966** | **0.9964** | **4.7022** |
| **RowGabriel** | **0.9972** | **0.9972** | **4.1898** | **RowGabriel** | **0.9966** | **0.9964** | **4.7022** |
| EM-AMMI0 | 0.4241 | -3.9672 | 175.0750 | EM-AMMI0 | 0.3909 | -5.3135 | 197.3799 |
| EM-AMMI1 | 0.0013 | -1240.88 | 2768.28 | EM-AMMI1 | 0.0029 | -741.76 | 2140.88 |
|  | Missing 10% and Outliers = 4% | | |  | Missing 20% and Outliers = 4% | | |
| GabrielEigen | 0.4258 | -18.9240 | 350.6361 | GabrielEigen | 0.3127 | -25.4553 | 404.0409 |
| TwoStagesG | 0.9967 | 0.9967 | 4.5153 | TwoStagesG | 0.9964 | 0.9961 | 4.8935 |
| QuartileG | 0.9971 | 0.9971 | 4.2237 | QuartileG | 0.9965 | 0.9963 | 4.7876 |
| **ColGabriel** | **0.9972** | **0.9972** | **4.1833** | **ColGabriel** | **0.9965** | **0.9964** | **4.7406** |
| **RowGabriel** | **0.9972** | **0.9972** | **4.1833** | **RowGabriel** | **0.9965** | **0.9964** | **4.7406** |
| EM-AMMI0 | 0.4223 | -16.9000 | 332.3496 | EM-AMMI0 | 0.4057 | -15.0201 | 314.4136 |
| EM-AMMI1 | 0.0072 | -8166.98 | 7099.476 | EM-AMMI1 | 0.0016 | -4033.33 | 4989.47 |

Table S5: Cross-validation study in Farias (2005)

|  | Dataset: Farias (2005) | | | | | | |
| --- | --- | --- | --- | --- | --- | --- | --- |
| Methods | Missing 10% and Outliers = 0% | | | Methods | Missing 20% and Outliers = 0% | | |
|  | ${Cos}^{2}$ | ${GF}_{1}$ | $P_{e}$ |  | ${Cos}^{2}$ | ${GF}_{1}$ | $P_{e}$ |
| **GabrielEigen** | **0.9715** | **0.9703** | **645.8117** | GabrielEigen | 0.9594 | 0.9555 | 790.6284 |
| TwoStagesG | 0.9715 | 0.9699 | 650.1834 | TwoStagesG | 0.9596 | 0.9554 | 792.2844 |
| **QuartileG** | **0.9715** | **0.9703** | **645.8117** | **QuartileG** | **0.9594** | **0.9558** | **788.7201** |
| ColGabriel | 0.9716 | 0.9702 | 647.5505 | ColGabriel | 0.9597 | 0.9555 | 790.5537 |
| RowGabriel | 0.9715 | 0.9701 | 648.1801 | RowGabriel | 0.9595 | 0.9552 | 793.7234 |
| EM-AMMI0 | 0.9714 | 0.9703 | 646.5387 | EM-AMMI0 | 0.9593 | 0.9555 | 791.0524 |
| EM-AMMI1 | 0.9550 | 0.9523 | 818.8332 | EM-AMMI1 | 0.9545 | 0.9508 | 831.9699 |
|  | Missing 10% and Outliers = 2% | | |  | Missing 20% and Outliers = 2% | | |
| GabrielEigen | 0.2525 | -3179478 | 6685883 | GabrielEigen | 0.2077 | -3229351 | 6738117 |
| TwoStagesG | 0.9714 | 0.9697 | 652.2607 | TwoStagesG | 0.9598 | 0.9552 | 793.6656 |
| **QuartileG** | **0.9714** | **0.9702** | **646.7809** | **QuartileG** | **0.9594** | **0.9557** | **789.1575** |
| ColGabriel | 0.9714 | 0.9702 | 646.7817 | ColGabriel | 0.9593 | 0.9554 | 791.8073 |
| **RowGabriel** | **0.9714** | **0.9702** | **646.7809** | RowGabriel | 0.9593 | 0.9554 | 791.8073 |
| EM-AMMI0 | 0.1377 | -4803014 | 8217463 | EM-AMMI0 | 0.1130 | -4362089 | 7831196 |
| EM-AMMI1 | 0.0029 | -1091759829 | 123892311 | EM-AMMI1 | 0.0012 | -698598081 | 99104755 |
|  | Missing 10% and Outliers = 4% | | |  | Missing 20% and Outliers = 4% | | |
| GabrielEigen | 0.2985 | -7769814 | 10451681 | GabrielEigen | 0.2358 | -8261684 | 10777428 |
| TwoStagesG | 0.9715 | 0.9695 | 655.2908 | TwoStagesG | 0.9593 | 0.9541 | 802.9930 |
| **QuartileG** | **0.9713** | **0.9701** | **648.4088** | **QuartileG** | **0.9592** | **0.9552** | **793.7071** |
| **ColGabriel** | **0.9713** | **0.9701** | **648.4088** | **ColGabriel** | **0.9592** | **0.9552** | **793.7071** |
| **RowGabriel** | **0.9713** | **0.9701** | **648.4088** | **RowGabriel** | **0.9592** | **0.9552** | **793.7071** |
| EM-AMMI0 | 0.2173 | -9700100 | 11678012 | EM-AMMI0 | 0.1704 | -8953735 | 11219744 |
| EM-AMMI1 | 0.0000 | -449556943 | 79501103 | EM-AMMI1 | 0.0008 | -323830143 | 67474463 |

Table S6: Cross-validation study in Filho et al. (2008)

|  | Dataset: Filho et al. (2008) | | | | | | |
| --- | --- | --- | --- | --- | --- | --- | --- |
| Methods | Missing 10% and Outliers = 0% | | | Methods | Missing 20% and Outliers = 0% | | |
|  | ${Cos}^{2}$ | ${GF}_{1}$ | $P_{e}$ |  | ${Cos}^{2}$ | ${GF}_{1}$ | $P_{e}$ |
| GabrielEigen | 0.9835 | 0.9833 | 508.3248 | GabrielEigen | 0.9626 | 0.9584 | 802.8300 |
| TwoStagesG | 0.9831 | 0.9827 | 517.2159 | TwoStagesG | 0.9624 | 0.9572 | 814.9169 |
| QuartileG | 0.9835 | 0.9833 | 508.3248 | QuartileG | 0.9626 | 0.9584 | 802.8300 |
| ColGabriel | 0.9834 | 0.9831 | 511.4664 | ColGabriel | 0.9627 | 0.9580 | 806.9431 |
| RowGabriel | 0.9836 | 0.9834 | 507.8226 | RowGabriel | 0.9628 | 0.9581 | 806.2172 |
| **EM-AMMI0** | **0.9837** | **0.9834** | **506.6919** | **EM-AMMI0** | **0.9637** | **0.9595** | **792.6663** |
| EM-AMMI1 | 0.9765 | 0.9754 | 617.1509 | EM-AMMI1 | 0.9512 | 0.9442 | 930.4732 |
|  | Missing 10% and Outliers = 2% | | |  | Missing 20% and Outliers = 2% | | |
| GabrielEigen | 0.1341 | -2476209 | 6196255 | GabrielEigen | 0.1174 | -2338166 | 6021065 |
| TwoStagesG | 0.9832 | 0.9828 | 516.8712 | TwoStagesG | 0.9627 | 0.9574 | 813.0653 |
| QuartileG | 0.9835 | 0.9833 | 508.4423 | QuartileG | 0.9624 | 0.9582 | 804.9173 |
| ColGabriel | 0.9833 | 0.9831 | 511.3288 | **ColGabriel** | **0.9625** | **0.9586** | **800.7563** |
| **RowGabriel** | **0.9835** | **0.9833** | **508.4205** | RowGabriel | 0.9624 | 0.9582 | 804.9173 |
| EM-AMMI0 | 0.1074 | -2122455 | 5736605 | EM-AMMI0 | 0.1109 | -1794034 | 5274133 |
| EM-AMMI1 | 0.0042 | -743505223 | 107368678 | EM-AMMI1 | 0.0022 | -444828335 | 83048504 |
|  | Missing 10% and Outliers = 4% | | |  | Missing 20% and Outliers = 4% | | |
| GabrielEigen | 0.2215 | -5610318 | 9326729 | GabrielEigen | 0.1886 | -5378890 | 9132338 |
| TwoStagesG | 0.9828 | 0.9822 | 524.7669 | TwoStagesG | 0.9624 | 0.9566 | 820.5064 |
| **QuartileG** | **0.9834** | **0.9832** | **509.9175** | **QuartileG** | **0.9622** | **0.9580** | **807.4555** |
| **ColGabriel** | **0.9834** | **0.9832** | **509.9175** | **ColGabriel** | **0.9622** | **0.9580** | **807.4555** |
| **RowGabriel** | **0.9834** | **0.9832** | **509.9175** | **RowGabriel** | **0.9622** | **0.9580** | **807.4555** |
| EM-AMMI0 | 0.2304 | -4135030 | 8007095 | EM-AMMI0 | 0.2101 | -3689975 | 7563927 |
| EM-AMMI1 | 0.0138 | -1064869366 | 128494270 | EM-AMMI1 | 0.0137 | -684866702 | 103047775 |

Table S7: Cross-validation study in Flores et al. (1998)

|  | Dataset: Flores et al. (1998) | | | | | | |
| --- | --- | --- | --- | --- | --- | --- | --- |
| Methods | Missing 10% and Outliers = 0% | | | Methods | Missing 20% and Outliers = 0% | | |
|  | ${Cos}^{2}$ | ${GF}_{1}$ | $P_{e}$ |  | ${Cos}^{2}$ | ${GF}_{1}$ | $P_{e}$ |
| GabrielEigen | 0.9872 | 0.9865 | 410.5341 | GabrielEigen | 0.9854 | 0.9838 | 449.1093 |
| TwoStagesG | 0.9863 | 0.9855 | 425.9257 | **TwoStagesG** | **0.9866** | **0.9855** | **425.2030** |
| QuartileG | 0.9862 | 0.9853 | 428.1143 | QuartileG | 0.9851 | 0.9838 | 449.9965 |
| ColGabriel | 0.9866 | 0.9859 | 418.7772 | ColGabriel | 0.9846 | 0.9831 | 459.0390 |
| RowGabriel | 0.9858 | 0.9847 | 437.2494 | RowGabriel | 0.9854 | 0.9838 | 449.6744 |
| **EM-AMMI0** | **0.9874** | **0.9867** | **407.7913** | EM-AMMI0 | 0.9868 | 0.9854 | 426.1380 |
| EM-AMMI1 | 0.9798 | 0.9791 | 510.4263 | EM-AMMI1 | 0.9807 | 0.9788 | 514.6047 |
|  | Missing 10% and Outliers = 2% | | |  | Missing 20% and Outliers = 2% | | |
| GabrielEigen | 0.0352 | -53790.79 | 819291.4 | GabrielEigen | 0.0408 | -89762.95 | 1058355 |
| TwoStagesG | 0.9866 | 0.9856 | 424.2914 | **TwoStagesG** | **0.9863** | **0.9851** | **431.9112** |
| QuartileG | 0.9861 | 0.9853 | 428.1917 | QuartileG | 0.9851 | 0.9839 | 447.8423 |
| ColGabriel | 0.9867 | 0.9859 | 418.8455 | ColGabriel | 0.9854 | 0.9839 | 448.4796 |
| **RowGabriel** | **0.9870** | **0.9863** | **413.1962** | RowGabriel | 0.9854 | 0.9840 | 447.3074 |
| EM-AMMI0 | 0.0753 | -50805.31 | 796231.3 | EM-AMMI0 | 0.0531 | -39037.2 | 697951.6 |
| EM-AMMI1 | 0.0009 | -6964728 | 9322503 | EM-AMMI1 | 0.0001 | -3357232 | 6472486 |
|  | Missing 10% and Outliers = 4% | | |  | Missing 20% and Outliers = 4% | | |
| GabrielEigen | 0.0727 | -219438.6 | 1654771 | GabrielEigen | 0.1445 | -37913.01 | 687828.6 |
| TwoStagesG | 0.9864 | 0.9852 | 429.1694 | **TwoStagesG** | **0.9865** | **0.9851** | **431.2744** |
| QuartileG | 0.9858 | 0.9849 | 434.2243 | QuartileG | 0.9848 | 0.9834 | 454.8712 |
| **ColGabriel** | **0.9867** | **0.9860** | **417.8281** | ColGabriel | 0.9854 | 0.9840 | 446.9893 |
| **RowGabriel** | **0.9867** | **0.9860** | **417.8281** | RowGabriel | 0.9853 | 0.9840 | 447.2648 |
| EM-AMMI0 | 0.2096 | -108062.7 | 1161236 | EM-AMMI0 | 0.1469 | -67768.33 | 919595.9 |
| EM-AMMI1 | 0.0074 | -32809036 | 20233787 | EM-AMMI1 | 0.0094 | -3862984 | 6942916 |

Table S8: Cross-validation study in Mattos et al. (2013)

|  | Dataset: Mattos et al. (2013) | | | | | | |
| --- | --- | --- | --- | --- | --- | --- | --- |
| Methods | Missing 10% and Outliers = 0% | | | Methods | Missing 20% and Outliers = 0% | | |
|  | ${Cos}^{2}$ | ${GF}_{1}$ | $P_{e}$ |  | ${Cos}^{2}$ | ${GF}_{1}$ | $P_{e}$ |
| GabrielEigen | 0.9815 | 0.9806 | 1.8454 | GabrielEigen | 0.9824 | 0.9796 | 1.8937 |
| **TwoStagesG** | **0.9839** | **0.9829** | **1.7336** | TwoStagesG | 0.9825 | 0.9798 | 1.8823 |
| QuartileG | 0.9805 | 0.9796 | 1.8917 | QuartileG | 0.9821 | 0.9798 | 1.8858 |
| ColGabriel | 0.9825 | 0.9813 | 1.8101 | ColGabriel | 0.9827 | 0.9798 | 1.8829 |
| RowGabriel | 0.9818 | 0.9810 | 1.8253 | **RowGabriel** | **0.9825** | **0.9800** | **1.8759** |
| EM-AMMI0 | 0.9815 | 0.9806 | 1.8476 | EM-AMMI0 | 0.9810 | 0.9784 | 1.9461 |
| EM-AMMI1 | 0.9305 | 0.9257 | 3.6115 | EM-AMMI1 | 0.9525 | 0.9441 | 3.1348 |
|  | Missing 10% and Outliers = 2% | | |  | Missing 20% and Outliers = 2% | | |
| GabrielEigen | 0.7114 | 0.3344 | 10.8124 | GabrielEigen | 0.8229 | 0.4719 | 9.6314 |
| **TwoStagesG** | **0.9826** | **0.9812** | **1.8149** | TwoStagesG | 0.9820 | 0.9786 | 1.9399 |
| QuartileG | 0.9817 | 0.9809 | 1.8319 | QuartileG | 0.9821 | 0.9795 | 1.8966 |
| ColGabriel | 0.9812 | 0.9801 | 1.8680 | **ColGabriel** | **0.9828** | **0.9798** | **1.8860** |
| RowGabriel | 0.9820 | 0.9812 | 1.8186 | **RowGabriel** | **0.9828** | **0.9798** | **1.8860** |
| EM-AMMI0 | 0.3264 | -3.1259 | 26.9210 | EM-AMMI0 | 0.2630 | -5.2950 | 33.2528 |
| EM-AMMI1 | 0.0171 | -47.9723 | 92.7481 | EM-AMMI1 | 0.0400 | -31.0529 | 75.0349 |
|  | Missing 10% and Outliers = 4% | | |  | Missing 20% and Outliers = 4% | | |
| GabrielEigen | 0.8105 | -0.7048 | 17.3050 | GabrielEigen | 0.8204 | -0.2008 | 14.5233 |
| TwoStagesG | 0.9827 | 0.9811 | 1.8236 | TwoStagesG | 0.9827 | 0.9786 | 1.9399 |
| **QuartileG** | **0.9822** | **0.9818** | **1.7897** | **QuartileG** | **0.9816** | **0.9791** | **1.9155** |
| ColGabriel | 0.9808 | 0.9799 | 1.8806 | ColGabriel | 0.9824 | 0.9787 | 1.9323 |
| RowGabriel | 0.6291 | 0.2501 | 11.4767 | RowGabriel | 0.6055 | -0.4106 | 15.7410 |
| EM-AMMI0 | 0.3789 | -5.3531 | 33.4060 | EM-AMMI0 | 0.3190 | -6.9068 | 37.2676 |
| EM-AMMI1 | 0.0024 | -611.6567 | 328.0489 | EM-AMMI1 | 0.0357 | -110.3381 | 139.8466 |

Table S9: Cross-validation study in Rad et al. (2013)

|  | Dataset: Rad et al. (2013) | | | | | | |
| --- | --- | --- | --- | --- | --- | --- | --- |
| Methods | Missing 10% and Outliers = 0% | | | Methods | Missing 20% and Outliers = 0% | | |
|  | ${Cos}^{2}$ | ${GF}_{1}$ | $P_{e}$ |  | ${Cos}^{2}$ | ${GF}_{1}$ | $P_{e}$ |
| **GabrielEigen** | **0.9843** | **0.9833** | **1.2006** | GabrielEigen | 0.9761 | 0.9726 | 1.5399 |
| TwoStagesG | 0.9564 | 0.9529 | 2.0183 | TwoStagesG | 0.9583 | 0.9511 | 2.0574 |
| QuartileG | 0.9781 | 0.9758 | 1.4456 | **QuartileG** | **0.9761** | **0.9726** | **1.5394** |
| ColGabriel | 0.9789 | 0.9766 | 1.4223 | ColGabriel | 0.9752 | 0.9708 | 1.5884 |
| RowGabriel | 0.9767 | 0.9739 | 1.5015 | RowGabriel | 0.9737 | 0.9686 | 1.6484 |
| EM-AMMI0 | 0.9724 | 0.9712 | 1.5789 | EM-AMMI0 | 0.9648 | 0.9596 | 1.8697 |
| EM-AMMI1 | 0.9628 | 0.9598 | 1.8656 | EM-AMMI1 | 0.9696 | 0.9657 | 1.7215 |
|  | Missing 10% and Outliers = 2% | | |  | Missing 20% and Outliers = 2% | | |
| GabrielEigen | 0.8052 | 0.3319 | 7.6025 | GabrielEigen | 0.8752 | 0.6595 | 5.4271 |
| TwoStagesG | 0.9581 | 0.9538 | 1.9981 | TwoStagesG | 0.9594 | 0.9520 | 2.0383 |
| QuartileG | 0.9782 | 0.9760 | 1.4411 | **QuartileG** | **0.9761** | **0.9725** | **1.5422** |
| **ColGabriel** | **0.9842** | **0.9832** | **1.2039** | ColGabriel | 0.9760 | 0.9725 | 1.5437 |
| RowGabriel | 0.9815 | 0.9800 | 1.3150 | RowGabriel | 0.9737 | 0.9692 | 1.6316 |
| EM-AMMI0 | 0.5277 | -0.8580 | 12.6780 | EM-AMMI0 | 0.7144 | 0.2965 | 7.8012 |
| EM-AMMI1 | 0.0001 | -728.2547 | 251.1713 | EM-AMMI1 | 0.0148 | -230.6024 | 141.5474 |
|  | Missing 10% and Outliers = 4% | | |  | Missing 20% and Outliers = 4% | | |
| GabrielEigen | 0.8750 | -0.5660 | 11.6392 | GabrielEigen | 0.9120 | 0.5248 | 6.4114 |
| TwoStagesG | 0.9583 | 0.9540 | 1.9951 | TwoStagesG | 0.9595 | 0.9520 | 2.0368 |
| QuartileG | 0.9782 | 0.9761 | 1.4370 | **QuartileG** | **0.9759** | **0.9724** | **1.5453** |
| **ColGabriel** | **0.9839** | **0.9829** | **1.2150** | ColGabriel | 0.9759 | 0.9723 | 1.5494 |
| RowGabriel | 0.9813 | 0.9798 | 1.3227 | RowGabriel | 0.9437 | 0.9225 | 2.5891 |
| EM-AMMI0 | 0.4992 | -3.0431 | 18.7020 | EM-AMMI0 | 0.6735 | -0.2213 | 10.2787 |
| EM-AMMI1 | 0.0156 | -819.1413 | 266.3635 | EM-AMMI1 | 0.0537 | -471.8442 | 202.2504 |

Table S10: Cross-validation study in Yang (2007)

|  | Dataset: Yang (2007) | | | | | | |
| --- | --- | --- | --- | --- | --- | --- | --- |
| Methods | Missing 10% and Outliers = 0% | | | Methods | Missing 20% and Outliers = 0% | | |
|  | ${Cos}^{2}$ | ${GF}_{1}$ | $P_{e}$ |  | ${Cos}^{2}$ | ${GF}_{1}$ | $P_{e}$ |
| GabrielEigen | 0.9894 | 0.9893 | 0.5821 | GabrielEigen | 0.9504 | 0.9409 | 1.3669 |
| TwoStagesG | 0.9630 | 0.9626 | 1.0878 | TwoStagesG | 0.9129 | 0.9105 | 1.6829 |
| QuartileG | 0.9893 | 0.9892 | 0.5833 | **QuartileG** | **0.9526** | **0.9458** | **1.3099** |
| ColGabriel | 0.9660 | 0.9657 | 1.0414 | ColGabriel | 0.9170 | 0.9148 | 1.6418 |
| RowGabriel | 0.9665 | 0.9660 | 1.0365 | RowGabriel | 0.9159 | 0.9136 | 1.6534 |
| **EM-AMMI0** | **0.9896** | **0.9895** | **0.5768** | EM-AMMI0 | 0.9502 | 0.9406 | 1.3711 |
| EM-AMMI1 | 0.9613 | 0.9607 | 1.1153 | EM-AMMI1 | 0.9284 | 0.9217 | 1.5735 |
|  | Missing 10% and Outliers = 2% | | |  | Missing 20% and Outliers = 2% | | |
| GabrielEigen | 0.3380 | -14.8897 | 22.4201 | GabrielEigen | 0.3250 | -16.2457 | 23.3571 |
| TwoStagesG | 0.9447 | 0.9436 | 1.3360 | TwoStagesG | 0.9131 | 0.9107 | 1.6808 |
| QuartileG | 0.9890 | 0.9890 | 0.5902 | **QuartileG** | **0.9516** | **0.9441** | **1.3294** |
| ColGabriel | 0.9892 | 0.9891 | 0.5861 | ColGabriel | 0.9513 | 0.9429 | 1.3443 |
| **RowGabriel** | **0.9893** | **0.9892** | **0.5845** | RowGabriel | 0.9500 | 0.9408 | 1.3688 |
| EM-AMMI0 | 0.2002 | -24.0061 | 28.1257 | EM-AMMI0 | 0.2150 | -23.4623 | 27.8181 |
| EM-AMMI1 | 0.0011 | -4040.0110 | 357.5399 | EM-AMMI1 | 0.0014 | -3554.3740 | 335.3684 |
|  | Missing 10% and Outliers = 4% | | |  | Missing 20% and Outliers = 4% | | |
| GabrielEigen | 0.3686 | -43.7786 | 37.6370 | GabrielEigen | 0.3617 | -42.7808 | 37.2153 |
| TwoStagesG | 0.9447 | 0.9436 | 1.3360 | TwoStagesG | 0.9133 | 0.9109 | 1.6784 |
| QuartileG | 0.9890 | 0.9889 | 0.5924 | QuartileG | 0.9504 | 0.9424 | 1.3495 |
| **ColGabriel** | **0.9892** | **0.9891** | **0.5870** | **ColGabriel** | **0.9510** | **0.9427** | **1.3466** |
| RowGabriel | 0.9892 | 0.9891 | 0.5882 | RowGabriel | 0.9497 | 0.9407 | 1.3695 |
| EM-AMMI0 | 0.1838 | -72.3638 | 48.1749 | EM-AMMI0 | 0.2214 | -62.4367 | 44.7971 |
| EM-AMMI1 | 0.0001 | -6889.1710 | 466.8684 | EM-AMMI1 | 0.0000 | -5674.7150 | 423.7305 |
